# Static Compaction on Coupled Precursors and Optimizing Molarity for Enhanced Strength and Durability of Geopolymer

**DOI:** 10.3390/ma17112509

**Published:** 2024-05-23

**Authors:** Khuram Rashid, Mounir Ltifi, Idrees Zafar, Muhammad Hashim Rafiqi, Muhammad Naeem Raoof

**Affiliations:** 1 Department of Architectural Engineering and Design, Faculty of Civil Engineering, University of Engineering and Technology, Lahore 54890, Pakistan; 2 Department of Civil Engineering, Imam Mohammad Ibn Saud Islamic University, Riyadh 11432, Saudi Arabia; mltifi@imamu.edu.sa; 3 Department of Civil Engineering, National Engineering School of Gabes, University of Gabes, Gabes 6072, Tunisia

**Keywords:** proportioning FA and slag, molarity and curing conditions, severe exposures, comparative analysis, feasibility

## Abstract

The static compaction technique emphasizes the reduced activator dosage required to develop geopolymers. Therefore, it is crucial to comprehend the optimal alkaline activator concentration for blending low-calcium precursor (fly ash) with high-calcium precursor (GGBS) to produce geopolymer blocks. This work was designed to optimize structural blocks’ compressive strength and durability. In experimentation, fly ash (FA) and slag (GGBS) proportions were initially investigated under NaOH solution with varying molarity (8–12) and curing conditions to develop a load-bearing structural block. Subsequently, the durability of the optimized block was evaluated over 56 days through subjection to sulfate and acidic solutions, with efflorescence monitored over the same period. The results reveal that the structural block comprised of 100% FA exhibits the highest compressive strength and lowest bulk density. Conversely, the block incorporating 25% slag that underwent hot curing demonstrates a remarkable 305% strength increase compared to ambient curing. Considering the physico-mechanical performance, the 100% FA block was chosen for durability investigation. The findings indicate a substantial strength loss exceeding 40% after exposure to sulfate and acidic environments over 56 days, coupled with pronounced efflorescence. Catastrophic failure occurs in all cases due to significant strength deterioration. The FTIR spectrum revealed the shifting of the wavenumber to a higher value and verified the depolymerization and leaching of alumina under acidic exposure. However, the developed geopolymer blocks demonstrate superior sustainability and feasibility compared to conventional fired clay bricks and cement-based FA bricks. Despite slightly higher costs, these blocks exhibit greater strength than their counterparts after enduring severe exposures.

## 1. Introduction

Bricks play a pivotal role in the global construction sector. Fired clay bricks are traditionally crafted from clay, but their production entails energy-intensive vitrification at around 1300 °C in kilns [1]. This process, as highlighted, is not only energy intensive but also results in a large amount of carbon dioxide (CO_2_) emissions due to calcination. As a result, finding alternatives to fired clay bricks is imperative. Alkali-activated materials, specifically geopolymers, are emerging as sustainable and environmentally friendly construction materials and present a feasible alternative to traditional bricks [2]. However, molding material is not a viable approach to meet the industrial demand for brick, but a recently developed pressing process may be used for geopolymers [3]. The lower activator dosage may reduce the physico-mechanical and durability performance, and therefore, it must be investigated thoroughly. 

Geopolymers, a subset of alkali-activated materials, are created through single- or two-part processes [4,5,6]. Their strength development is consistent, driven by the activation of alumina–silicate materials in an alkaline environment, leading to the formation of tetrahedral structures such as N-A-S-H (sodium alumina–silicate hydrate) and C-A-S-H (calcium alumina–silicate hydrate) gels. Geopolymers are formulated from either a low-calcium precursor like fly ash (FA) or high-calcium precursors such as ground granulated blast furnace slag (GGBS). Their compressive strength largely depends on the precursor’s amorphous silica and alumina content. FA dissolution occurs in high-molarity NaOH solutions at elevated temperatures, whereas slag dissolution is more effective under ambient conditions with lower NaOH concentrations [7]. This influences the choice of curing regime (ambient or hot) and duration [8]. Various factors affect geopolymer strength, including the alkaline to precursor ratio (A/P), the sodium silicate (SS) to sodium hydroxide (SH) ratio, SH molarity, and curing temperature. Research into the proportioning of FA and GGBS under different NaOH concentrations and curing regimes is crucial [6,9,10], as is the optimization of these parameters, given the lack of data on mechanical strength improvements through static compaction.

Compression casting, also known as pressure casting, requires less water and allows for immediate removal from the machine, facilitating dry or wet curing. In geopolymers, a hot pressing technology known as warm pressing [11] has been utilized for rapid production. This method involves pressing and curing geopolymers at elevated temperatures. Pressure levels of 20 and 40 MPa have proven suitable for developing structural blocks [12]. Studies have explored various combinations, such as coupling FA with sugarcane bagasse ash [13], clay [14], dune sand, and lime [3], to develop brick/structural blocks by employing static compaction. However, while precursor proportions were optimized in each study, researchers have yet to investigate the intermixing of a low-calcium precursor (FA) with a high-calcium precursor (GGBS) to create geopolymer blocks. Considering the reduced activator requirements of the pressing technique, determining the optimal concentration of NaOH solution and selecting appropriate curing conditions is paramount. A thorough investigation is warranted to address these inquiries.

Geopolymer durability is crucial, as this material often outperforms conventional cement-based resilient materials [15,16]. Deterioration primarily occurs from Na+ ion infiltration and exposure to sulfate solutions (e.g., Na_2_SO_4_ and MgSO_4_) [16], leading to vertical cracks and disruption of the aluminosilicate gel structure. This disruption cleaves –Si–O–Si– bonds and causes silicon leaching as pH levels increase [17], while sulfate attack prompts ettringite formation from C-S-H phases, reducing mechanical strength. These interactions result in linear declines in both compressive strength and Vickers hardness on exposed surfaces [18]. Enhancing sulfate resistance in Na_2_SiO_3_-activated GGBS/FA mixtures can be achieved by increasing the activator dosage, which reduces porosity and boosts compressive strength [19]. However, pressure catalysis limits dosage increases, potentially increasing deterioration risk and necessitating further investigation. FA-based geopolymers show higher resistance to acidic environments than OPC [15,16], thanks to a more resilient cross-linked aluminosilicate polymer structure [20]. Acid attacks depolymerize the aluminosilicate system and lead to zeolite formation [21], with ion penetration from acidic solutions causing aluminum leaching and forming silicic acid [9]. Trace calcium, potassium, magnesium, and iron amounts may also leach into the H_2_SO_4_ solution [22]. Over time, diffusing SO_4_^2−^ anions and counter-diffusing Ca^2+^ ions form gypsum crystals within the matrix [20,21,23]. Incorporating GGBS into FA-based geopolymers reduces acid resistance by forming C-A-S-H gel, which leads to gypsum formation in the corroded layer. In contrast, FA-based geopolymers that form only N-A-S-H gel exhibit superior strength and durability compared to those incorporating GGBS.

Efflorescence, identified as a physio-chemical process, involves the dissolution and diffusion of atmospheric CO_2_ into concrete, which then reacts with Ca^2+^ to precipitate calcium carbonate (CaCO_3_) on the surface [24]. In geopolymer-based materials, efflorescence primarily results in the formation of sodium carbonate (Na_2_CO_3_·nH_2_O) products, with occasional reports of sodium phosphate hydrate (Na_3_PO_4_⋅12H_2_O) formation [25,26]. Efflorescence in geopolymers is a significant concern due to water-soluble alkali ions in substantial quantities within the geopolymer matrix [27,28]. The efflorescence severity correlates with higher activator content, as excessive alkaline activator leaches to the surface in moist conditions, reacting with CO_2_ to create white precipitate sodium carbonate (Na_2_CO_3_) products on the surface. Earlier studies have demonstrated that the pore structure influences the movement and extraction of free alkalis during leaching, stemming from excess, weakly bonded, or unreacted alkalis. A denser and more intricate pore network impedes the migration of alkali cations [24,29,30].

The author’s research group did a series of work to develop a geopolymer-based structural block as an alternative to conventional fired clay brick. Initially, the FA and clay were used to develop a 50 mm cube, and their proportion was optimized to develop an alternative to fired clay brick [2]. An optimized proportion was used, and the influence of static compaction was investigated [14]. Afterward, the influence of molding pressure, curing temperature, and time on the physico-mechanical performance of FA-based brick was investigated [12]. Recently, a one-part binder was developed, and its application as a paving block was investigated. The parameters that influence the compressive strength of FA-based geopolymer were also investigated by analyzing a comprehensive database and employing artificial intelligence modules. This work was further extended using the optimized parameters and tuning them for FA- and GGBS-based geopolymers. In addition to the compressive strength, the durability of the developed block was also investigated.

This research focuses on developing structural blocks that align with industrial standards, utilizing static compaction as the primary fabrication method. The study initially evaluates the physico-mechanical properties of these blocks, exploring a range of variables, including different mix ratios of FA and GGBS; varying concentrations of sodium hydroxide (NaOH) solutions at 8, 10, and 12 molarity; and different curing conditions—either ambient or heated. After establishing the baseline mechanical properties, the study identifies which block compositions exhibit the best physico-mechanical performance. These optimized block specimens are then subjected to rigorous durability tests. This involves immersing the blocks in solutions of magnesium sulfate (MgSO_4_) and sulfuric acid (H_2_SO_4_) for a duration of 56 days to evaluate their resistance to chemical degradation. Concurrently, the same timeframe is used to assess the degree of efflorescence formation on these specimens, which can be a common problem affecting masonry products’ aesthetic and structural integrity. The final phase of the research compares the mechanical strength and durability of the newly developed geopolymer-based structural blocks against those of commercially available conventional fired clay bricks and cement-based FA bricks. This comparison is crucial not only in assessing the blocks’ performance but also in evaluating their sustainability and economic feasibility for immediate practical applications. The goal is to determine whether these geopolymer-based blocks provide a viable, cost-effective alternative to traditional building materials in the construction industry.

## 2. Materials and Methods

### 2.1. Materials

**Figure 1:**
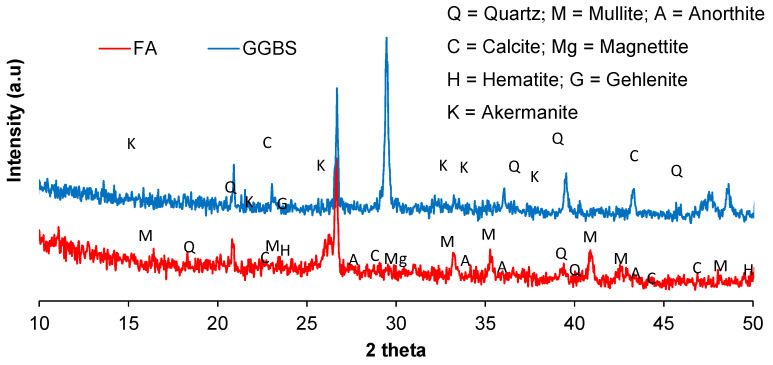
Diffractogram of FA and GGBS used in this study.

In this study, FA and GGBS were utilized as aluminosilicate material sources (precursors). The FA was derived from a coal-based thermal power plant in Sahiwal, Pakistan, while GGBS was sourced from the Nukshi Star Slag Industry in Mirpur, AJK, Pakistan. Chemical composition analysis of both FA and GGBS was conducted through XRF analysis, with the resulting values detailed in Table 1. It illustrates that FA is the low-calcium precursor, and GGBS is a high-calcium precursor. Along with that, both have enough quantity of alumina and silicate to develop C-A-S-H and N-A-S-H gels in geopolymerization. The XRD analysis of FA and GGBS presented in Figure 1 and the mineralogy of both precursors is also highlighted. Figure 1 illustrates that the quartz peaks are fewer and may have amorphous content.

As the fine aggregate, river sand was employed, having a fineness modulus value of 1.8. Alkaline activation was achieved using a combination of sodium silicate (Na_2_SiO_3_) and sodium hydroxide (NaOH). The sodium hydroxide pellets (99% purity) were acquired from Sitara Chemicals Karachi. In the laboratory, NaOH solutions with varying molarities (8M, 10M, and 12M) were prepared using distilled water. Na_2_SiO_3_ was in viscous liquid form, its viscosity was 60 cP, and its modulus (SiO_2_/Na_2_O) was 1.2.

To assess durability, exposure conditions were established using MgSO_4_ and H_2_SO_4_. Solutions of both MgSO_4_ and H_2_SO_4_ at a concentration of 5% weight/volume ratio were prepared in the laboratory.

### 2.2. Specimen Preparation

A 1:1 proportion of precursor (FA) and sand was placed into a bucket and dry mixed for 3 min using a mechanical mixer. Following this, the wet mixing process was initiated by introducing alkaline activators. To achieve a semi-wet paste/matrix, the liquid to solid ratio (*l*/*s*) or the ratio of alkaline activators to solids was set at 0.25 after several trials. This (*l*/*s*) ratio was fixed by employing the flowability test on the fresh past according to ASTM C1437 [31]. It was also confirmed after demolding that no bulging was observed after immediate demolding. The Na_2_SiO_3_ solution was combined with the NaOH solution at a ratio of 2.5.

**Figure 2:**
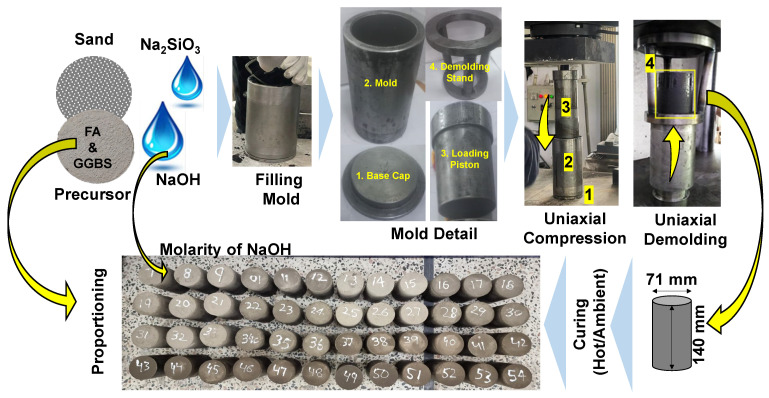
The specimen preparation and optimizing parameters considered in this work to develop structural blocks by static compaction.

Once the semi-wet mixture was prepared, cylindrical specimens were cast by applying a pressure of 20 MPa with the assistance of a universal testing machine. Swift demolding was executed immediately using the same universal testing machine, carefully transitioning the cylindrical mold. The mold possessed a diameter of 71 mm and a height of 150 mm. For each distinct mix design, three specimens underwent curing at room temperature. At the same time, another three were cured at 70 °C in an electric oven for 24 h, followed by subsequent curing at room temperature. The schematic procedure of the specimen preparation is illustrated in Figure 2. The procedure was repeated, varying the molarity of the NaOH solution in increments of 8, 10, and 12. Additionally, at these three molarities, the quantity of FA was adjusted, and GGBS was introduced. Various combinations of FA with GGBS were explored, including ratios of 100/0, 75/25, 50/50, and 25/75. The comprehensive details of all mixture proportions and acronyms utilized in this study are elucidated in Table 2.

### 2.3. Testing

#### 2.3.1. Physico-Mechanical Performance

The physico-mechanical performance of the specimens was assessed by conducting compressive strength and bulk density tests. The examination of compressive strength involved adhering to ASTM C39 protocols [32], wherein cylindrical specimens were tested after 7 days of curing. Equation (1) was used to calculate compressive strength ($f_{c}^{\prime }$), given as the ratio of ultimate load and area under the loading. The average value of three specimens is reported in this study. The bulk density is measured as the weight to volume ratio, as given in Equation (2).

(1) $$f_{c}^{\prime }=\frac{Load}{Area}$$

(2) $$Bulk\;density=\frac{Weight\;}{Volume}$$

#### 2.3.2. Durability Performance

**Figure 3:**
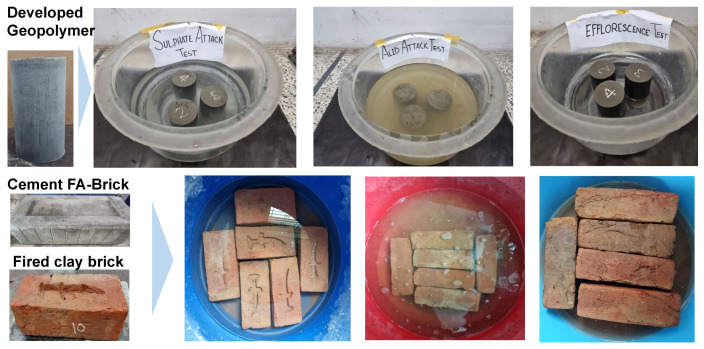
Durability performance investigation of developed geopolymer block and commercially available cement–FA brick and fired clay brick.

A series of tests were conducted to evaluate the durability of geopolymer specimens, including resistance to sulfate attack, acid attack, and efflorescence. The resistance of geopolymer specimens to a sulfate solution was investigated by immersing them in a solution containing 50 g of MgSO_4_ per liter of water, adhering to ASTM C 1012 guidelines [33]. The specimens’ residual strength was evaluated after immersions lasting 7 days, 28 days, and 56 days. It was compared with the strength of the specimen that was not immersed and termed as a “control” specimen in this work. The durability investigation is presented in Figure 3. The mass before and after immersion in the solution was also measured by weighing the specimens.

For exploring the specimens’ resistance to acid attack, the specimens were submerged in a solution of H_2_SO_4_ (Figure 3) with a 5% weight/volume ratio, and following these immersions, compressive strength tests were conducted at intervals of 7 days, 28 days, and 56 days.

The efflorescence test necessitated immersing specimens to a depth equivalent to one-third of their height in water, maintaining consistent water levels every 24 h (Figure 3). Visual assessments were performed at 7 days, 28 days, and 56 days to gauge the extent of efflorescence coverage on the specimens.

The durability test was performed on commercially available fired clay bricks and FA-incorporated cement-based bricks. The same exposure condition was applied (Figure 3), and performance was evaluated for up to 28 days. Finally, based on the physico-mechanical and durability performance, the feasibility of the developed geopolymer-based brick was explored, considering the influence of cost.

### 2.4. Sustainability Quantification

The sustainability of the developed block was evaluated by considering three domains of sustainability: societal, economic, and environmental factors. The compressive strength and durability performance make the block societally acceptable. Economically, the cost of the developed structural block was calculated. The cost of the components was adopted from the market rate system of Pakistan and converted to dollars by using a conversion rate of 285 and tabulated in Table 3.

The third pillar of sustainability is the environmental aspect, and thus, CO_2_ emissions were quantified for the developed block. The quantities of material are enlisted in Table 3. However, CO_2_ is also emitted during heat curing, and the following Equation (3) was used to quantify it [36].

(3) $${{CO}_{2}\;emission}_{\left(heating+curing\right)}=\left(\frac{75\;{}^{\circ}C\times 0.039\;t-{CO}_{2}/m^{3}}{85\;{}^{\circ}C}\right)$$

## 3. Results and Discussions

### 3.1. Optimizing Compressive Strength

The investigation focused on the compressive strength of the developed geopolymer specimens. This study analyzed the strength variation based on factors such as the concentration of NaOH solution, the curing conditions, and the proportion of precursors (FA and GGBS). The pressure catalysis approach was employed for this analysis, and the detailed discussion is given in the following sub-sections.

#### 3.1.1. Impact of Molarity under Ambient Curing Conditions

**Figure 4:**
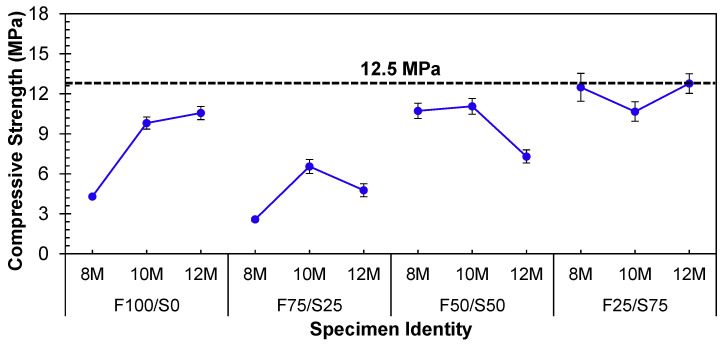
Influence of molarities of NaOH solution on the strength of different combinations of FA–GGBS under ambient conditions.

From Figure 4, it was noted that the strength of the geopolymer specimens increased as the concentration of the NaOH solution increased for FA-based specimens. This rise in concentration influenced the alkaline nature of the geopolymer mixture, thereby impacting the rate of reaction kinetics and the final characteristics of the geopolymer material [37]. Generally, a higher concentration of NaOH resulted in a more alkaline environment, facilitating a quicker and more comprehensive geopolymerization reaction [4]. This heightened reactivity led to accelerated strength development and, subsequently, higher compressive strength [5,38]. 

Precursors with lower calcium content, like FA, require a higher concentration of NaOH. Consequently, the strength of the geopolymer composed entirely of FA (F100/S0) exhibited an upward trend as the concentration of NaOH solution increased from 8 to 12, as depicted in Figure 4.

On the contrary, GGBS serves as a high-calcium precursor and exhibits heightened reactivity under ambient conditions. Consequently, a lower concentration of NaOH solution is advised. Excessive alkalinity, on the other hand, can trigger the rapid setting of the paste, leading to compromised workability and potentially diminished long-term strength. Thus, by combining precursors (FA and GGBS), a nearly equivalent strength was accomplished using 8 and 10 molar NaOH solutions, as demonstrated in the F50/S50 specimen (Figure 4). However, the highest strength achievable under ambient conditions, varying proportions of precursors, and different concentrations of NaOH remained limited to only 12.5 MPa. This level of strength needs to be improved for applications requiring structural integrity.

#### 3.1.2. Impact of Molarity under Ambient Curing Conditions

**Figure 5:**
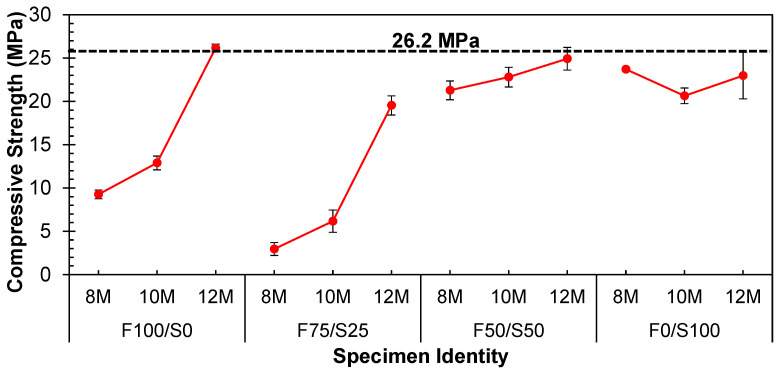
Influence of molarities of NaOH solution on the strength of different combinations of FA–GGBS under hot curing conditions.

It is worth noting that the curing conditions play a pivotal role in accelerating reactivity, leading to swift geopolymerization and consequent strength improvement [37]. When dealing with FA-based geopolymers, a high-temperature curing method is recommended to achieve early-age strength (within 7 days). Although comparable strength levels can be attained under ambient conditions, this requires a significantly extended duration [6]. In contrast, GGBS achieves early strength even under ambient conditions. The impact of various NaOH molarities and different precursor proportions on the compressive strength of specimens subjected to hot curing is depicted in Figure 5. The findings reveal that the highest strength (26.2 MPa) was achieved in the case of the 100% FA precursor (F100/S0) specimen using a 12 molar NaOH solution. A similar trend was observed for the F75/S25 specimens, although the compressive strength was less against all molarities compared to F100/S0 specimens. However, by substituting FA with GGBS (F50/S50 and F0/S100 specimens), almost similar strength was observed under all molarities of NaOH solution (Figure 5), and it was also comparable to the strength of the F100/S0 specimen at 12 molar concentration of NaOH solution. Additionally, the dominant strength performance was observed at a 12 molar concentration of NaOH solution among all the different molar concentrations.

#### 3.1.3. Comparison between Curing Conditions

**Figure 6:**
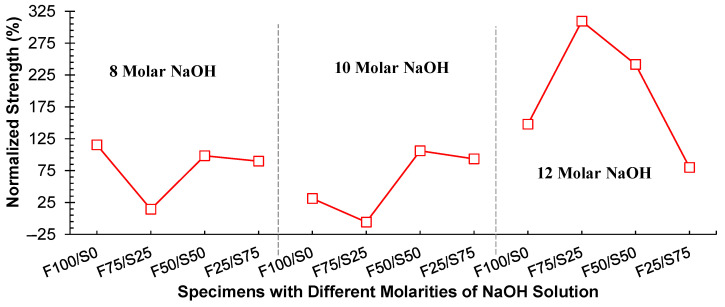
Comparison of ambient and hot curing conditions on the strength of geopolymer block.

A comparison was also made between the strength achieved under ambient conditions and the strength attained through hot curing. This distinction in strength enhancement due to hot curing was quantified by normalizing strength with ambient condition, and it is visually represented in Figure 6. It was observed that the most substantial increase in strength was achieved when employing a 12-molar concentration of NaOH solution, with a quantified improvement exceeding 300%. While considering only FA (F100/S0) as the precursor, the strength increase through hot curing was 148%, and with partial substitution of 25% FA with GGBS, the strength increase reached 309%. However, as the proportion of GGBS was further increased, the magnitude of strength increases diminished, going only up to 80%. The main reason for the strength under hot curing is the dissolution of Al and Si from the FA, which increases with the increase in the temperature, and beyond 65 °C, there was significant dissolution of Al and Si, and the process was completed in almost 24 h [39]. For high-calcium precursors such as GGBS, leaching of Al and Si is observed in alkaline environments at ambient conditions, with a reduction observed at high temperatures.

Furthermore, Figure 6 also indicates that for other NaOH molarities (8 and 10 molars), there was a notable increase in strength when employing hot curing, although this increase remained below 100%. In conclusion, it can be inferred that a 12-molar concentration of NaOH solution is the optimal choice for both FA-based geopolymer systems and when coupled with 25% GGBS. Nonetheless, increasing the proportion of GGBS beyond this threshold diminishes its potential for strength improvement.

### 3.2. Bulk Density

**Figure 7:**
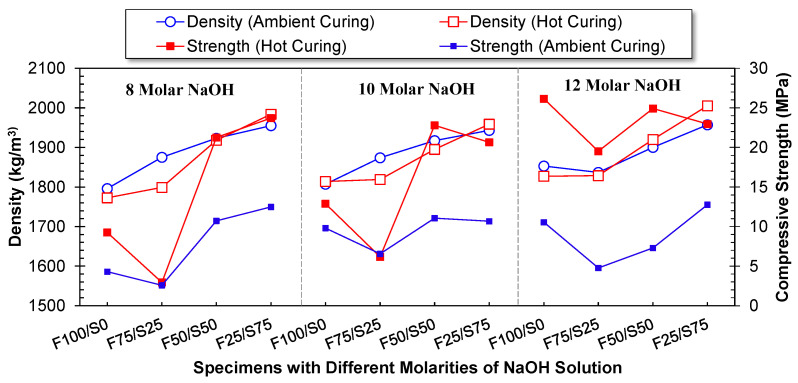
Influence of FA/GGBS and molarity of NaOH solution on the bulk density and compressive strength of geopolymer-based structural block.

The comparison between the densities of geopolymer specimens cured under ambient conditions and those subjected to hot curing across various NaOH solution molarities and different precursor proportions is displayed in Figure 7. Notably, geopolymer specimens based on FA exhibited the lowest densities among all tested NaOH solution molarities. The impact of curing, whether ambient or hot, on density was relatively minimal, with almost identical densities observed between the two curing methods. However, an increase in the proportion of GGBS leads to a linear increase in density. The compressive strength values are also plotted in Figure 7. It was observed that the trend is almost the same as that observed for bulk density, although the compressive strength at ambient conditions is significantly less. 

This phenomenon can be attributed to the inherent bulk density of GGBS being higher than that of FA. Nevertheless, the incremental rise in density due to increased GGBS content was moderate. The recorded minimum density was 1773 kg/m^3^, while the maximum density reached 2005 kg/m^3^. This is noteworthy, as the developed geopolymer-based blocks weigh less than traditional fired clay bricks, which possess a density of around 2000 kg/m^3^. Furthermore, geopolymer blocks demonstrate significant weight reduction compared to conventional concrete, with a density of approximately 2400 kg/m^3^. This emphasizes the potential of geopolymer materials for applications where reduced weight is advantageous.

### 3.3. Optimizing Specimen Based on Physico-Mechanical Performance

**Figure 8:**
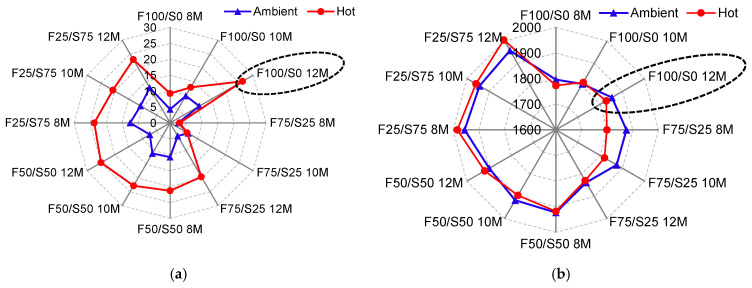
Influence of FA/GGBS and molarity of NaOH solution on the (**a**) compressive strength and (**b**) bulk density of geopolymer-based structural.

Figure 8a presents the comprehensive summary of all specimens’ compressive strength, including both curing conditions. It was observed that ambient curing condition is not viable for geopolymer structural applications, and maximum strength is obtained against hot curing conditions. The first thing that was optimized was the hot curing condition, and the next was the molarity of the NaOH solution and the proportioning of FA–GGBS. From Figure 8a, it can be easily visualized that a specimen with 100% FA (F100/S0) at a 12-molar concentration has maximum strength. Therefore, only one specimen, “F100/S0” or the FA-based specimen, was optimized based on the strength value.

Figure 8b presents the comprehensive summary of the bulk density of all specimens under both curing conditions. The variation in the bulk density value under both curing conditions was observed to be marginal. However, the sample with 100% FA has the lowest value, specifically at a 12-molar concentration of NaOH solution and under hot curing conditions. Therefore, based on physico-mechanical performance, an F100/S0 specimen at the 12-molar concentration of NaOH solution and hot curing condition was used to develop a structural block, and it was further investigated for durability performance, as illustrated in the following Section 3.4.

### 3.4. Durability of Structural Block

The specimen with 100% FA (F100/S0) was optimized, as discussed in detail in Section 3.3. The durability of the optimized specimen was investigated by exposing it to sulfate and acid attacks. The efflorescence of the block was also assessed at different ages, and a detailed discussion is given in the following sub-sections.

#### 3.4.1. Sulfate Attack

**Figure 9:**
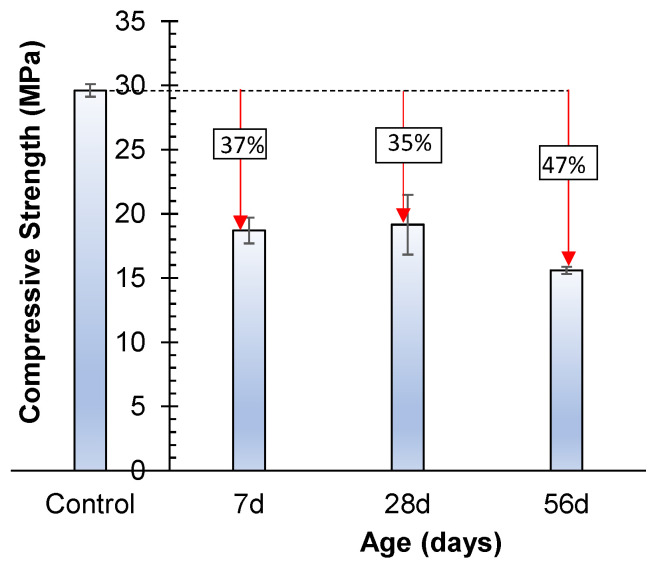
Influence of sulfate attack on the compressive strength of FA-based geopolymer structural block.

The resistance of geopolymer specimens to the sulfate solutions was investigated at different ages. The compressive strength of all the geopolymer specimens was decreased when exposed to the 5% concentration of MgSO_4_ solution, and a summary of the results is shown in Figure 9. About 36% decrease in compressive strength was observed for the geopolymer specimens at 7 days’ and 28 days’ exposure in comparison to the control specimens. The significant loss at 7 days was due to the reaction of sulfate ions (SO_4_^2−^) with the unreacted aluminosilicate material (FA). The concentration of unreacted FA may be higher in the developed geopolymer block due to a smaller amount of alkaline activator given by the pressure catalysis approach [12]. Therefore, a significant reduction was at 7 days, and the same reduction was observed after 28 days of exposure. However, after exposure for 56 days to MgSO_4_ solution, the reduction in strength was 47% of the actual strength.

The reduction may be due to the formation of secondary mineral phases, such as ettringite or thaumasite and gypsum, which are expansive compounds and can cause internal stresses and damage to the geopolymer matrix. The other reason for loss in compressive strength is due to the decomposition of C-A-S-H gel. The mechanism of the strength loss can be explained in terms of the disappearance of Si–O–T (T = Si or Al) in the C-A-S-H phase, and the resulting product formed was gypsum [15]. In this work, FTIR analysis was performed, and respective curves are explained in Section 3.4.4.

The other reason for the reduction in strength is the presence of sulfate in the FA, as revealed from XRF analysis Table 1. Excess sulfate present in the precursor reacts with other components of the geopolymer matrix, leading to the formation of expansive compounds and subsequent damage. The formation of expansive compounds in the geopolymer matrix causes the specimens to expand, resulting in cracks on the surface of geopolymer specimens. Due to the presence of these cracks, sulfate ingression phenomena occur, and a considerable reduction in compressive strength is observed. In addition to the physical damage caused by expansion, sulfate attack can also result in weight loss or gain in the geopolymer material. The formation of secondary minerals during sulfate attack can lead to the leaching of soluble components from the geopolymer matrix. This leaching can result in the loss of mass from the material. On the other hand, in some cases, the reaction between sulfates and the geopolymer can lead to the formation of additional solid phases, which can contribute to weight gain. The precipitation of new mineral phases can increase the overall mass of the material. This shows the influence of sulfate attack on the mass gain/loss of the geopolymer specimens. Approximately 0.5%, 1%, and 1.5% increases in the mass of geopolymer specimens were observed at 7 days, 28 days, and 56 days, respectively. The water absorbed during this process is the reason behind the mass gain. During the deterioration of the specimens, the porosity of the specimens might be increased due to the decomposition of specimens during sulfate attack reaction mechanism, and water retention in the pores of the specimens increases with time, causing the mass gain.

#### 3.4.2. Acid Attack

**Figure 10:**
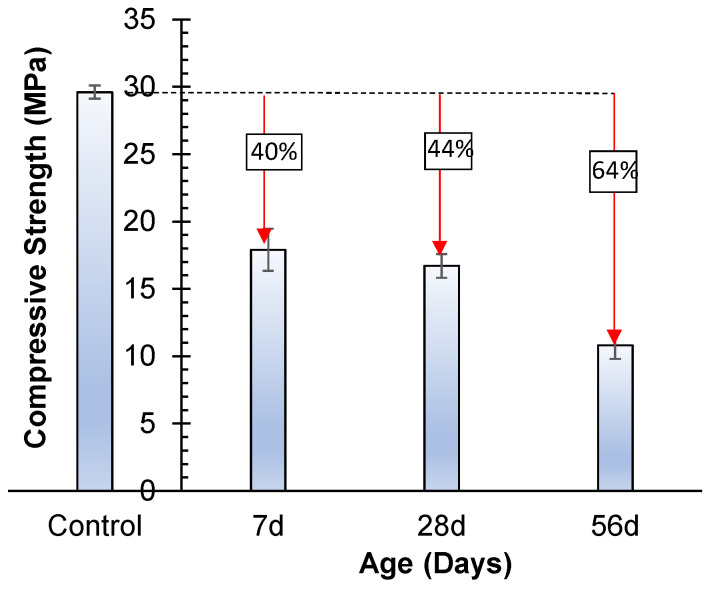
Influence of under-acid attack on compressive strength.

The mechanical performance of geopolymer specimens was analyzed under an acidic environment. The resistance of geopolymer specimens against the 5% solution of H_2_SO_4_ was investigated. The geopolymer specimens were cured for 56 days before exposure to the acidic medium. Approximately 40%, 44%, and 64% reductions in compressive strength were observed for the geopolymer specimens immersed for 7 days, 28 days, and 56 days, respectively (Figure 10). Considerable decrease in compressive strength was observed in a study of some geopolymers prepared with a mixture of Na_2_SiO_3_ and NaOH solution or KOH solution [21]. The reduction in compressive strength is due to the depolymerization of alumino-silicate polymers in low-pH environments and the formation of zeolites [21].

The weight of geopolymer specimens increased after immersion in the H_2_SO_4_ solution for 7 days and 28 days. Quantitatively, 0.7% and 2.6% increases in the mass of specimens were observed for 7 days and 28 days, respectively. The increase in the mass of specimens might be due to the deterioration of the surface and the formation of pores. These pores facilitate water penetration. As the saturated mass of specimens was determined before immersion in acidic media, the formation of pores increases the saturated mass. Therefore, the specimens gained mass after 7 days and 28 days of immersion. However, the mass of the specimens decreased by 2.6% after 56-day immersion in H_2_SO_4_ solution, which may be due to the addition of solid phases developed by the reaction of acid with geopolymers. The precipitation of new mineral phases can increase the overall mass of the material. The same phenomena of deterioration of the specimen surface and cracks in the specimens can explain this. The retention capacity of specimens decreased. Therefore, the mass of specimens decreased.

#### 3.4.3. Efflorescence

**Figure 11:**
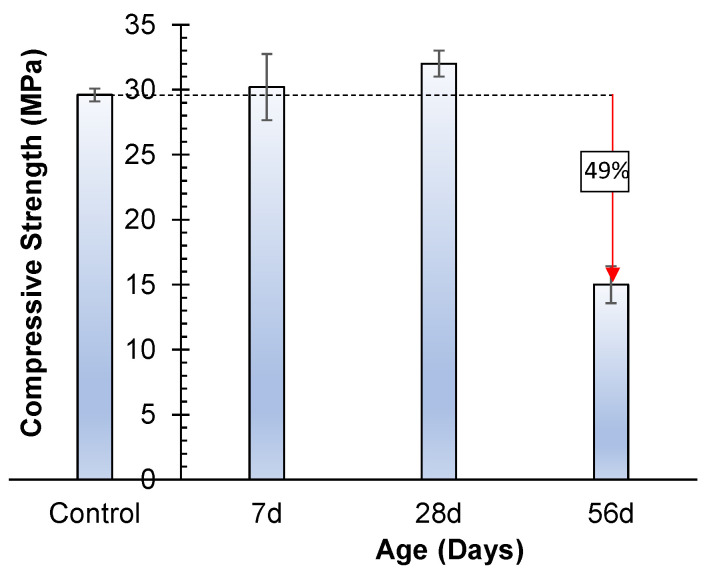
Influence of efflorescence on compressive strength.

The impact of efflorescence on both the compressive strength and visual characteristics of geopolymer specimens was explored across various time intervals. The geopolymer’s compressive strength demonstrated an increase of 2% at 7 days and 8% at 28 days, as indicated by the results of compressive strength tests (see Figure 11). However, a substantial reduction of 49% in compressive strength was observed at the 56-day mark. The ages of the geopolymer specimens tested for compressive strength were 63 days, 84 days, and 112 days, corresponding to their respective points at 7 days, 28 days, and 56 days.

**Figure 12:**
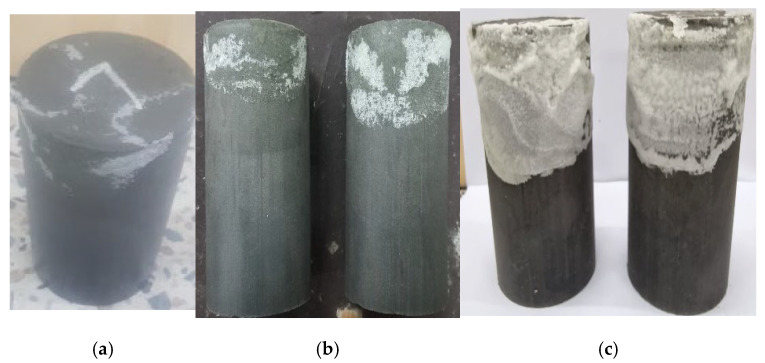
Visual efflorescence in geopolymer specimens at (**a**) 7 days, (**b**) 28 days, and (**c**) 56 days.

Upon immersion of the geopolymer specimens in water, minimal signs of efflorescence were discernible at 7 days and 28 days post immersion (see Figure 12). The slight enhancement in compressive strength at these intervals might be attributed to the extended curing of the geopolymer specimens, which were initially aged for 56 days before undergoing the durability test. This age difference of 56 days between the geopolymer specimens used for the durability test and their corresponding control specimens (with actual strength) likely contributed to this effect.

However, after the 56-day immersion period, a significant amount of efflorescence became apparent, covering approximately 30% to 40% of the specimen surfaces (see Figure 12). This presence of efflorescence corresponded to a reduction of 49% in compressive strength. While efflorescence in fired clay bricks is usually considered an aesthetic concern, its impact on the mechanical properties of geopolymer became pronounced when studied over an extended duration [40]. The occurrence of efflorescence in geopolymers can be attributed to sodium carbonate salts, whereas in products based on OPC, efflorescence is linked to calcium carbonates [37].

#### 3.4.4. FTIR Curves

**Figure 13:**
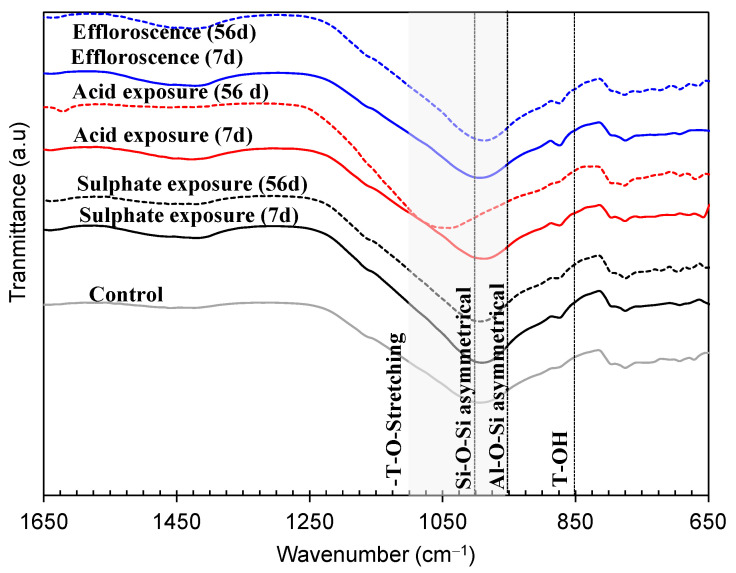
Comparison of FTIR curves of FA-based geopolymer under sulfate attack at different ages.

The FTIR analysis was carried out after the durability investigations were performed. The spectrum of 7 d and 56 exposures was selected, and the corresponding FTIR spectrum is presented in Figure 13. A comparison was made among all the control specimens. T-OH is presented at 850 cm^−1^ wavelengths. The asymmetrical Al-O-Si and Si-O-Si are presented by 960 and 1000 cm^−1^, respectively. The shaded region in Figure 13 presents the 960–1100 T-O stretching range.

In the case of sulfate exposure, it was observed that there was no significant shifting of the wavenumber of Al-O-Si and Si-O-Si. However, the depth was varied and may influence the rate of geopolymerization, as explained in [2]. But, in the case of acidic exposure, significant shifting of the wavenumber was observed, thus verifying the depolymerization. The acidic ions disrupt the –Si–O–Si– and –Si–O–Al– bonds, causing aluminum leaching and silicic acid. Such deterioration can also be verified by significant strength loss after 56 days of exposure among all specimens, i.e., 64%. However, the efflorescence case is very similar to the exposure of sulfate attack, and no significant variation in the FTIR spectrum was observed.

#### 3.4.5. Failure Modes

**Figure 14:**
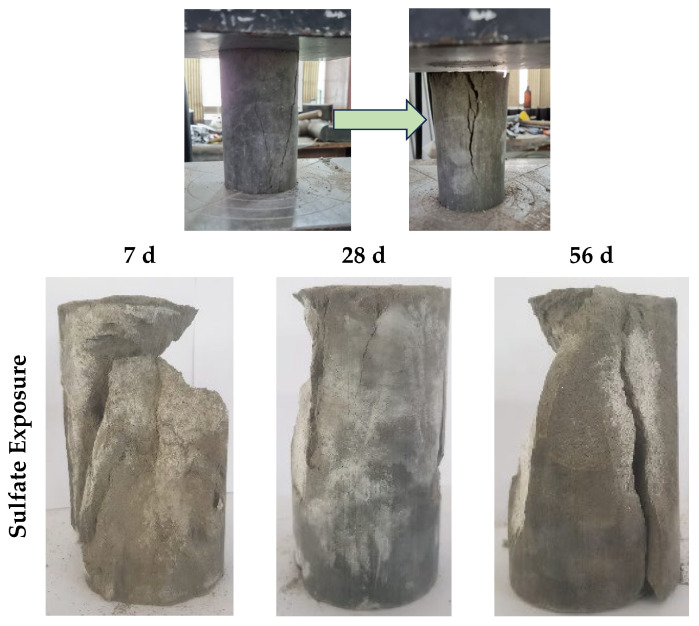

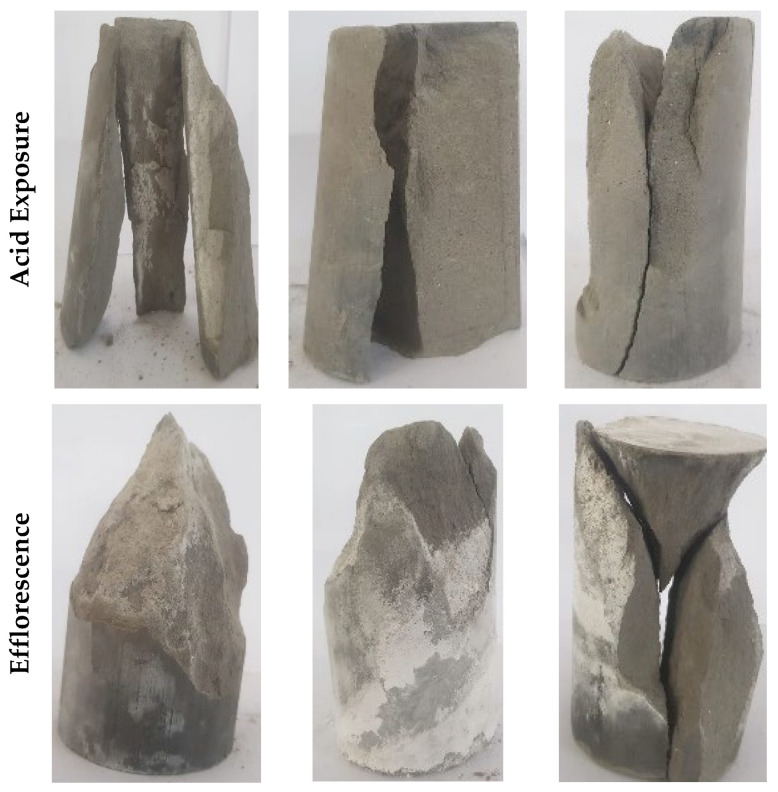
Failure modes of specimens at different ages with different exposure conditions.

Figure 14 illustrates the different ways in which specimens were affected by sulfate attack, acid attack, and efflorescence. The nature of these failures is categorized as catastrophic. This is evident from the separation of layers within the specimens, which can be attributed to a substantial decrease in their compressive strength. Detailed discussions regarding these occurrences are found in Section 3.4.1, Section 3.4.2, Section 3.4.3 and Section 3.4.4.

In the context of sulfate attack, when exposed to MgSO_4_ for 56 days, the specimen fractured into two distinct halves due to compressive failure. Similarly, when subjected to acidic conditions, the specimens exhibited splitting at all stages, indicating a significant reduction in strength, exceeding 40%. However, during the initial 7 and 28 days of efflorescence exposure, the failure pattern followed conventional lines. This absence of splitting can be attributed to a slight increase in compressive strength during these periods. Nevertheless, by the 56-day mark, the failure pattern transitioned to a catastrophic mode due to notable loss in strength, causing a decrease in the overall structural integrity of the specimen.

### 3.5. Comparative Analysis with Commercial Bricks

**Figure 15:**
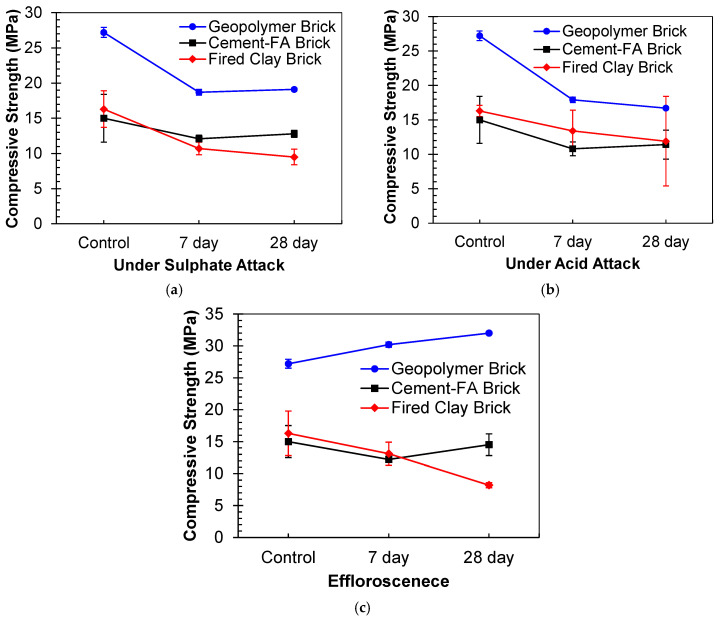
Comparative analysis of masonry blocks against durability performance: (**a**) sulfate exposure, (**b**) acid exposure, and (**c**) partial exposure to water.

In the developing nations of Asia, fired clay bricks have been extensively employed for masonry purposes. However, a recent development involves the introduction and commercial utilization of new fly ash-based cement bricks (referred to as cement–FA bricks) in various projects. The durability of these commercially available bricks has also been subjected to investigation, and their performance has been compared with that of newly developed geopolymer-based structural blocks. A comparison of the durability among these three types of bricks is depicted in Figure 15.

It has been noted that the geopolymer bricks exhibit superior performance when exposed to MgSO_4_ conditions. Although there is a more pronounced initial strength reduction within the first 7 days in the geopolymer bricks, as depicted in Figure 15a, their strength at both 7 and 28 days surpasses that of the other commercially available brick types. A similar pattern emerges for exposure to acidic conditions, as shown in Figure 15b. While the reduction trend in strength across the three brick types remains nearly identical during the 7–28-day exposure period, a substantial strength reduction is evident in the geopolymer-based specimens at 7 days. However, even in this case, the geopolymer bricks still outperform their counterparts and thus present a more viable option compared to conventionally utilized bricks.

Interestingly, a different trend is observed when considering the effect of efflorescence, as portrayed in Figure 15c. A slight decrease in compressive strength is witnessed in all three brick types. Notably, the geopolymer-based bricks exhibit an increase in strength, while marginal strength reduction is observed in the other brick types. Ordinarily, efflorescence negatively impacts the aesthetic appearance of buildings, and fired clay bricks display inadequate resistance against this phenomenon.

### 3.6. Sustainability and Feasibility Assessment of Developed Structural Block

For acceptance, compressive strength and durability performance are the main criteria. Therefore, the control strength and performance under sulfate and acid attacks opted to quantify this domain. The bulk density of the developed structural block is 1840 kg/m^3^ (Figure 7) as explained in Section 3.2. The proportion of FA/sand is 1:1, that with A/P is 0.25 (Section 2.2), and the material used for 1 m^3^ is also calculated in Table 3. Regarding the cost (per kg) and quantity of components (for 1 m^3^), the cost for 1 m^3^ is USD 34.7. By considering the block size of 225 × 115 × 75 mm, which is widely used in the construction industry for brick, the cost of one thousand bricks is USD 67.4.

The CO_2_ emissions for the 1 m^3^ material is 75.84 kg of CO_2_, and for the developed block for size 225 × 115 × 75 mm, the CO_2_ emission is 0.15 kg of CO_2_ emissions. This calculation considers only material-based CO_2_ emissions, and CO_2_ from heat curing was also incorporated from Equation (3). Therefore, the total CO_2_ emissions for the developed block is 0.21 kg-CO_2_ per block. The CO_2_ emitted for the fired clay-based brick is 0.592, which is significantly more, and the temperature required for manufacturing fired clay brick is about 1350 °C; therefore, such difference is obvious. For cement-based brick, the large amount of CO_2_ is emitted due to cement content.

**Figure 16:**
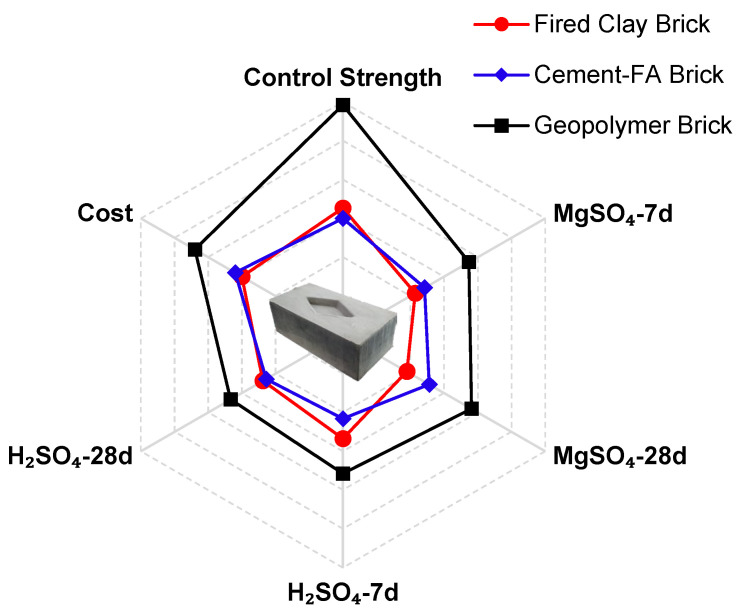
Comparative analysis and feasibility of the developed geopolymer-based bricks.

The feasibility of the developed geopolymer block was assessed by comparing up to 28 days of durability performance with conventional fired clay brick and cement–FA brick. Along with durability performance, the cost and CO_2_ emissions were also calculated for geopolymer brick and compared with the commercial price of the other two types of bricks. All results are summarized in Table 4 and compared graphically in Figure 16. It can be observed that the performance of newly developed geopolymer brick surpasses the other brick types in all aspects. Its cost is also higher, as depicted in Figure 16. 

As it is made in the laboratory, the cost of an alkaline activator is a little bit higher compared to purchasing small quantities from the local market. However, after the industrialization of geopolymers, its cost can obviously be reduced and made comparable to the other commercially available products if the economic index is made by normalizing the cost with respect to the strength. It can be noted that the economic index of geopolymer-based brick is lower than the other types of brick and concluded as more feasible for actual application.

## 4. Conclusions

This work was designed to develop a geopolymer-based structural block alternative to conventional fired clay bricks and cement-based FA bricks. Initially, the compressive strength was optimized by varying the proportions of FA and GGBS, molarity of the NaOH solution, and curing conditions. The optimized specimen was tested for durability and compared with other conventional bricks, and finally, the feasibility of the developed product was determined. The following conclusions were drawn from this experiment:

1. Higher molarity yielded an increase in the strength of the FA-based geopolymer, but when coupled with GGBS, higher molarity is detrimental to the strength. This is due to the leaching of Al and Si at different concentrations from low- (FA) and high- (GGBS) calcium precursors;
2. Compressive strength was optimized using 100% FA as a precursor and a 12-molar concentration of NaOH solution to develop a geopolymer structural block by employing a pressure catalysis approach and curing at hot conditions. The value was 26.2 MPa. However, the strength was increased three times when shifted from ambient curing to hot curing conditions with the 75% FA and 25% GGBS specimen;
3. The density of the FA-based specimen has the least bulk density (1773 kg/m^3^), and by increasing the proportion of GGBS, the bulk density increases linearly and reaches up to 2000 kg/m^3^. However, the influence of the molarity of NaOH solution and curing condition has a marginal effect on the bulk density;
4. Both sulfate and acidic exposure have a detrimental influence on the strength of geopolymer, and more than 40% reduction in strength was observed when exposed for 56 days. The acidic exposure is very damaging, and the reduction in strength is up to 64%;
5. The strength was increased up to 28 days by partially dipping in water, and no efflorescence was observed, whereas at 56 days, significantly salt crystalline formations appeared, and strength was also decreased by 49%;
6. The failure mode was catastrophic, and layer splitting was observed after failure. This was aligned with the significant loss in the reduction in the strength of the specimens;
7. The durability performance was also compared with conventional commercially available fired clay brick and cement–FA-based brick. The developed brick had better performance up to 28 days of investigation. However, its cost is higher, and it may have a lower economic index and seem more feasible than conventional materials. It can be considered an alternative to traditional masonry blocks.

## Figures and Tables

**Table 1:** XRF analysis of FA and GGBS used in this work.

Material	Oxides (%)								LOI
	SiO_2_	Al_2_O_3_	CaO	Fe_2_O_3_	MgO	K_2_O	Na_2_O	SO_3_	(%)
FA	56.3	23.1	9.0	6.4	1.7	0.6	0.3	---	<3.0
GGBS	37.4	13.3	40.9	1.3	1.6	---	0.4	0.6	

**Table 2:** Summary of mix designs used in this work.

Sr. No	FA (%)	GGBS (%)	Sand (%)	NaOH Molarity	Acronyms
1	50	0	50	8	F100/S0
2	50	0	50	10	F100/S0
3	50	0	50	12	F100/S0
4	37.5	12.5	50	8	F75/S25
5	37.5	12.5	50	10	F75/S25
6	37.5	12.5	50	12	F75/S25
7	25	25	50	8	F50/S50
8	25	25	50	10	F50/S50
9	25	25	50	12	F50/S50
10	12.5	37.5	50	8	F25/S75
11	12.5	37.5	50	10	F25/S75
12	12.5	37.5	50	12	F25/S75

**Table 3:** Cost and CO_2_ emission of developed structural block.

Sr. No	Material	Cost (USD Per Ton)	Quantity for 1 m^3^	CO_2_ Emission Per Ton	Ref. **
1	Fly ash	8.8	817.7	19.6	[34]
2	Sand	2.5	817.7	1.3	[34]
3	Na_2_SiO_3_	122.8	146	237	[35]
4	NaOH	350.9	58.4 *	1120	[34]

* 21.56 kg for 12 Molar NaOH solution; ** references for CO_2_ emissions.

**Table 4:** Summary of performance of sustainability of bricks.

Description	Fired Clay Brick	Cement–FA Brick	Geopolymer Brick
Control strength	16.3	15	29.6
MgSO_4_—7 d	10.7	12.1	18.7
MgSO_4_—28 d	9.5	12.8	19.1
H_2_SO_4_—7 d	13.4	10.8	17.9
H_2_SO_4_—28 d	11.9	11.4	16.7
Cost (USD per 1k)	52.6	56.1	67.4
CO_2_ emissions	0.549	0.3	0.21

## Data Availability

Data are contained within the article.
